# An Error Estimation System for Close-Range Photogrammetric Systems and Algorithms †

**DOI:** 10.3390/s23249715

**Published:** 2023-12-08

**Authors:** Anton Poroykov, Olga Pechinskaya, Ekaterina Shmatko, Danil Eremin, Nikita Sivov

**Affiliations:** Moscow Power Engineering Institute, National Research University, Krasnokazarmennaya Str., 14, 111250 Moscow, Russia; zhukovaov@mpei.ru (O.P.); shmatkoyv@mpei.ru (E.S.); yeremindv@mpei.ru (D.E.); sivovny@mpei.ru (N.S.)

**Keywords:** close-range photogrammetry, error estimation, physical modelling, fringe projection profilometry, phasogrammetry, image pattern correlation technique

## Abstract

Close-range photogrammetry methods are widely used for non-contact and accurate measurements of surface shapes. These methods are based on calculating the three-dimensional coordinates of an object from two-dimensional images using special digital processing algorithms. Due to the relatively complex measurement principle, the accurate estimation of the photogrammetric measurement error is a non-trivial task. Typically, theoretical estimations or computer modelling are used to solve this problem. However, these approaches cannot provide an accurate estimate because it is impossible to consider all factors that influence the measurement results. To solve this problem, we propose the use of physical modelling. The measurement results from the photogrammetric system under test were compared with the results of a more accurate reference measurement method. This comparison allowed the error to be estimated under controlled conditions. The test object was a flexible surface whose shape could vary smoothly over a wide range. The estimation of the measurement accuracy for a large number of different surface shapes allows us to obtain new results that are difficult to obtain using standard approaches. To implement the proposed approach, a laboratory system for the error estimation of close-range photogrammetric measurements was developed. The paper contains a detailed description of the developed system and the proposed technique for a comparison of the measurement results. The error in the reference method, which was chosen to be phasogrammetry, was evaluated experimentally. Experimental testing of the stereo photogrammetric system was performed according to the proposed technique. The obtained results show that the proposed technique can reveal dependencies that may not be detected by standard approaches.

## 1. Introduction

The development of digital image capture devices and image processing algorithms has led to the development of a wide range of methods for measuring the geometric dimensions of various objects from their images. Such optical inspection methods are known as photogrammetry [1]. Historically, photogrammetry originated in the mapping of terrain from images taken by various aircraft [2]. The term close-range photogrammetry is used to measure objects of smaller sizes and at shorter distances [3].

Photogrammetric methods are based on determining the three-dimensional coordinates of a point in space from a two-dimensional image of it. In general, this determination is impossible without additional information. Different photogrammetric methods use various approaches to obtain this information. Three methods can be distinguished as the most common: the use of two (or more) video cameras (stereo photogrammetry), structured illumination together with a video camera (fringe projection profilometry and similar), and special marks with known sizes and/or positions (ArUco markers and others).

Photogrammetric methods have been widely used in several fields. Simple systems based on disparity calculations and stereo images are used for navigation tasks in robotics [4,5,6]. More complex systems with higher positional accuracy requirements can be used in medicine [7,8]. Increased requirements for measurement errors can be imposed in mechanical testing of parts and materials [9,10,11]. The widespread use of smartphones and the rapid development of digital video cameras have led to the development of specific methods for applying them to photogrammetry tasks [12,13,14]. The scales of measurements using close-range photogrammetric systems range from nano- [15] and microscale [16] to large scales [17,18].

Photogrammetric measurements in the aerospace industry, including flight tests [17,19,20,21], can be described separately. Such measurements are performed under specific conditions: a small tilt angle between the optical axis and the observation plane, a high level of vibration, and difficult conditions such as strong natural illumination. Therefore, it is particularly important to determine the influence of these factors on measurement error.

All measurement methods were evaluated for errors. This is necessary when designing new photogrammetric systems and developing new image-processing algorithms, as well as when testing existing ones. Two main approaches are commonly used for error estimation. The first is based on mathematical or computational models. From these, theoretical formulas were derived, and computer modelling was performed to estimate the measurement error. One of the main approaches for photogrammetric methods is the estimation of measurement uncertainty through stereo vision calibration and measurement algorithms. This approach was developed in several studies [22,23]. This technique was further developed by applying the Monte Carlo method to simulate a large amount of input data to estimate the propagation of measurement uncertainty [24,25,26,27]. The classical approach to error estimation by processing synthesized images was presented in [28].

The second approach relies on experimental measurements. Usually, it uses either special test targets with known geometric dimensions or moves relative to the camera over known distances, or it uses some other measurement method with a higher accuracy than that of the test method or system. In [29], experimental studies were conducted to confirm the theoretical evaluation of [23]. In [20], the effects of vibration-induced changes in the effective values of stereo system calibration parameters during flight tests were investigated. In [30], the effect of camera calibration on the measurement results of a stereo photogrammetric system was investigated. For industrial applications, the experimental evaluation of the measurement accuracy was also investigated in [31]. In [32], the theoretical and experimental error estimation of the Digital Image Correlation (DIC) method for the angle between the cameras of a stereo system is given. An experimental comparison of stereophotogrammetric and interferometric measurement results is given in [33]. It is also worth mentioning experimental studies on the accuracy of industrially produced RGB-D cameras [34,35], which have recently been widely used in various fields ranging from medicine to security. In [13], a comparison of the accuracy of photogrammetric measurements of aperture and surface roughness of a rock fracture using a professional SLR camera and smartphone cameras was presented.

Despite the large number of studies on theoretical error estimation, it is difficult to consider all the conditions under which measurements are made and all the specificities of certain algorithms used in image processing. Experimental evaluation is mainly aimed at determining the error of specific measurement systems and algorithms, or at confirming the results of theoretical work. Most of this work uses test objects or translators to move flat objects at known distances. The size of the test objects, usually ceramic spheres with known dimensions, is much smaller than the field of view of a photogrammetric system. Flat objects moved by the translators represent objects that are too simple to measure. Neither option allows us to fully determine the error of the systems under test, taking into account possible nonlinearity and the whole measurement volume with statistical reliability.

This study attempted to develop a technique for error estimation by measuring a large number of random surfaces. The surfaces should cover most of the field of view of the photogrammetric system, and their shapes should be sufficiently complex to imitate the shape of real measured objects. It is difficult to produce a large number of test objects similar to ceramic spheres. Therefore, it is proposed that their shape be measured using a reference method, the error of which should be lower than that of the test method. Such a technique will allow us to obtain new error estimation results for a wide range of tested systems, which are inaccessible under the existing approaches.

This paper describes a hardware–software system for measurement error estimation in close-range photogrammetric systems and algorithms, implementing the proposed technique. The system uses a specially developed device for generating random surfaces: a deformable surface imitator, an optical distance sensor, and a phasogrammetric system for reference measurements. The surface measurement method using the phasogrammetric system and a comparison of the measurement results from the reference and tested methods are described in detail. The results of the experimental verification of the developed system using a granite test slab are presented. The verification results show that the instrumental error of the developed system is approximately 50 µm. A stereophotogrammetric system was tested by measuring 100 random surfaces, as an example of the application of the developed system. The results of the comparison over a series of measurements helped to reveal the increase in the measurement error in the center of the surface, which was not noticeable in individual surface measurements. The paper concludes with a discussion of the obtained results and an evaluation of the strengths and weaknesses of the proposed technique and the error estimation system created. Possible ways to improve the system and plans for future studies are also provided.

## 2. Materials and Methods

### 2.1. System for Estimating Photogrammetric Errors

Physical modelling has been proposed to estimate the error of close-range photogrammetric systems. The modelling technique involves measuring the sample surface using two methods. The first method was the tested one, for which error estimation was performed. The second method is the reference method, which provides a higher accuracy than the first method. The error of the test method was defined as the difference between the measurement results of the test and the reference methods. The shape of the sample surface must change between measurements. Obtaining an error estimate for a set of measurements allows statistically significant results.

**Figure 1:**
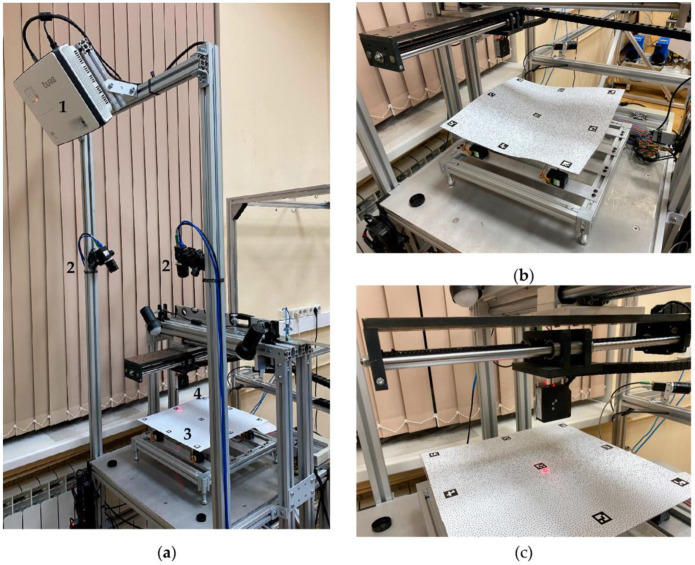
Error estimation system for close-range photogrammetric systems and algorithms: (**a**) general view of the system (1—projector, 2—photogrammetric system cameras, 3—imitator of the deformable surface, 4—optical distance sensor); (**b**) imitator of the deformable surface; (**c**) optical distance sensor.

To implement the described physical modelling, a measurement system [36] consisting of a deformable surface imitator and an optical distance sensor was previously developed (Figure 1a). The laser sensor was measured locally at a single point. In order to measure the shape of the entire surface, it was moved by linear actuators with stepper motors in a plane in two orthogonal directions.

The deformable surface imitator consisted of an aluminum base attached to digital servos. Each servo was rigidly connected to a flexible plate with a surface area of 380 × 380 mm^2^ located above the servos. This acts as a deformable surface. The total number of servos was eight (up to 16 can be connected), and they were evenly distributed on the surface of the plate. Changing the position of the servo causes proportional vertical displacement in the plate section above it. Each servo could move the plate section above it by a distance of ±25 mm. The appearance of the deformable surface imitator is shown in Figure 1b.

A Dongbu (Seoul, Republic of Korea) Herxulex DRS-0101 digital servo machine was used to shape the surface of the imitator. Each servo has two connectors, one input and one output. This allows all the simulator’s servos to be connected to a single cable. The power supply is 7–12 V. The position of the servos was set via a full-duplex UART serial interface with a maximum speed of 0.677 Mb/s. Connection to a computer is achieved via the WinChipHead (Nanjing, China) RS232-USB adapter, which provides a virtual COM port for the transmission of control commands. The control is implemented using device-specific binary commands with support for checking data integrity.

The optical distance sensor used was an LS5-40/50 laser triangulation sensor manufactured by SPE PRIZMA (Ekaterinburg, Russia). The sensor measured the distance to the deformable surface with an accuracy of 0.001 mm and an error of less than ±0.075 mm in the distance range of 50 mm, and the closest distance to the sensor was 40 mm. The spot size of the sensor laser beam at the center of the measurement range was 175 µm and did not exceed 300 µm over the entire range. The laser triangulation sensor is shown in Figure 1c.

The optical distance sensor was connected to a computer via an Profilic (Taipei, China) RS-485 interface using a USB adapter, which provided a virtual COM port for transmitting the control commands. The measurement results were received through device-specific ASCII messages. The sensor was supplied with a 12 V voltage. The movement of the sensor over the surface was performed using the Purelogic (Voronezh, Russia) linear motion modules of a CNC machine with stepper motors. The stepper motors were controlled using a Purelogic PLC545 controller. The control signals were transmitted via the standard STEP/DIR/ENABLE protocol from a Raspberry (Cambridge, UK) Pi2 single-board computer with PyCNC software [37] installed. The movement of the sensor was controlled by the computer via the Ethernet interface using the XML-RPC library.

The laser sensor presents advantages, such as a simple interpretation of the measurement results and known instrumental error. However, the movement of the sensor across the measured surface requires a considerable amount of time, which is directly proportional to the number of measured points, and induces unwanted vibrations. Consequently, an alternative method was used as a reference to measure the surface. The method must fulfil certain requirements for surface shape measurement, including non-contact capability with high accuracy and relative speed. It is also important that surface characteristics do not have a strong influence on the measurement results.

These requirements substantially limit possible options. Only optical methods meet all requirements. Interferometric and photogrammetric methods are promising optical techniques. Although interferometric systems offer high accuracy and speed, their implementation can be challenging and require costly optical components. One significant drawback is that the size of the optical elements is directly dependent on the size of the surface being measured when inspecting flat and convex surfaces [38]. Additionally, the air path section between the interferometer and measured object may experience vibrations and phase inhomogeneities, reducing the reliability of the interference methods [39,40]. Hence, photogrammetric methods, namely those using structured light, were chosen.

### 2.2. Phasogrammetric Measurement Method

Compared with methods using stereo image pairs, these methods reduce the dependence of the measurement accuracy on the surface character and simplify the processing algorithms. Fringe Projection Profilometry (FPP) methods are widely used. They are based on projecting special patterns (images) onto the measured surface in the form of a set of periodically repeating fringes with different intensity distributions. These methods are based on the calculation of the phase of the periodic signal present in the pattern with fringes. The patterns were projected onto the measured surface using a projector, and depending on the shape of the surface, the appearance of the fringe pattern changed. Thus, information about the surface shape is related to the phase of the periodic signal in the images of the projected pattern. Phase calculation from the images was performed using different algorithms depending on the intensity distribution used in the fringes. A comprehensive review of FPP methods is presented in [41,42].

The main criterion for selecting a reference method for the proposed method was high measurement accuracy. In [43,44,45], it was shown that FPP methods have the required accuracy. A value of less than 1 µm was reported as an estimate for these methods [45]. The standard 4-step algorithm for Phase Shift Profilometry (PSP) with sinusoidal fringes was chosen for phase-field acquisition in the created system. This method allows higher accuracy to be obtained compared to Fourier Transform Profilometry (FTP), whereas image processing is performed using simple mathematical expressions [42]. The disadvantages are low relative speed, as it requires the projection of four images to calculate one phase field, and instability to displacement of the measured objects. The latter is not considered, as the developing system measures a stationary surface.

The multifrequency (hierarchical) temporal phase unwrapping method [46] was chosen for phase recovery. The first projected pattern contains only one fringe (period); therefore, the recovered phase does not contain discontinuities. In the subsequent patterns, the number of periods gradually increases, leading to discontinuities in the phase fields. These discontinuities were recovered using the phase-field calculated from the first template.

The three-dimensional point cloud of the measured surface was reconstructed using phasogrammetry method [44,47]. This is a mixture of stereo vision and phase-shift profilometry. It uses reconstructed phase fields instead of images to find the corresponding points for the two video cameras. Phasogrammetry is less sensitive to intensity nonlinearities in experimental images and has a higher accuracy than other methods in the family. An additional advantage of phasogrammetry is its ability to autocalibrate the cameras based on the measurement results [44].

To measure the surface, a series of patterns is created, whose intensity is determined using the following formulas:

(1) Ih,ix,y=0.5+0.5·cos2π·f·xw−δi,Iv,  ix,y=0.5+0.5·cos2π·f·yh−δi,

where *I_h_* and *I_v_*—patterns for determining the horizontal and vertical phase fields; *x*, *y*—pixel coordinates of the created pattern; *i*—pattern number in the series for one frequency; *f*—fringe frequency; *w* and *h*—width and height of the pattern to be created; and *δ_i_*—phase difference introduced between fringes with the same frequency determined using the formula:

(2) $$\delta_{i}=2\cdot \pi /n\cdot i,$$

where *n*—number of steps required for the phase shifting. The minimum number of steps is 3, and in our work, we use a number of steps equal to 4.

The created patterns were projected onto the measured surface using a projector, and two cameras captured the images of the surface. The intensity of the captured images for each camera is described as follows:

(3) fir,c=Ar,c+Br,c·cosφr, c−2π·in,

where *f*—image intensity; *r*, *c*—pixel coordinates in the image; *A*(*r*, *c*)—the average intensity equivalent to the image captured under uniform illumination; *B*(*r*, *c*)—the intensity modulation related to pattern modulation and is proportional to the surface reflectivity; and *φ*(*r*, *c*)—the phase of the distorted fringe containing the depth information of the object surface.

To recover the phase for the n-step phase shift, we can use the formula in the general form:

(4) φr, c=tan−1∑i=0nfir,c·sin2π·i/n∑i=0nfir,c·cos2π·i/n.

The phase recovered calculated using this formula has discontinuities owing to the periodicity of trigonometric functions. To obtain a wider dynamic range and recover discontinuities in the phase field, hierarchical temporal phase unwrapping was used. For this purpose, several series of patterns with different frequencies, *f*, are projected onto the surface. The first series has a frequency of *f* = 1, contains only one fringe, and therefore has no phase discontinuity. To obtain the phase fields without phase discontinuity for frequencies other than 1, the following formula is used:

(5) $$\varphi_{u}\left(r,\;c\right)=\varphi \left(r,\;c\right)+2\pi \cdot k\left(r,c\right),$$

where *φ_u_*(*r*, *c*)—recovered phase without discontinuities; and *k*—an integer determined using the following formula:

(6) kjr,c=Roundfjfj−1·φj−1r, c−φjr, c2π,

where *j*—phase field number in the hierarchical series ($f_{0}=1$); and Round—rounding operator to an integer.

After processing the hierarchical series for the vertical and horizontal fringes, we obtained two phase fields ${\varphi_{u,\;v}}_{1,2}\left(r,\;c\right)$ and ${\varphi_{u,h}}_{1,2}\left(r,\;c\right)$ for each camera. The search for corresponding points for phase fields from different cameras was carried out according to the condition:

(7) $$\left\{\begin{array}{c} {\varphi_{u,\;v}}_{1}\left(r_{1},\;c_{1}\right)={\varphi_{u,\;v}}_{2}\left(r_{2},\;c_{2}\right)\\ {\varphi_{u,\;h}}_{1}\left(r_{1},\;c_{1}\right)={\varphi_{u,\;h}}_{2}\left(r_{2},\;c_{2}\right) \end{array}\right..$$

The search can be performed in various ways. In our work, we use the calculation of a two-dimensional look-up table (LUT), whose vertical and horizontal indices correspond to the vertical and horizontal phase values for the second camera, and each cell contains the pixel coordinates for the second camera with these phase values. For a preliminary estimate of the position of the corresponding point, the nearest point was searched using the phase values from the LUT cell. Then, the coordinates of the corresponding point are searched with a sub-pixel resolution based on the bilinear interpolation of the phase field. The two-dimensional point coordinates obtained for the two cameras were then used to calculate the three-dimensional points using the standard triangulation function from the OpenCV library.

The phasogrammetric image processing algorithm was implemented, in the form of software [48], in Python 3.9 using the Numpy 1.23.1, Scipy 1.8.1, and OpenCV 4.6.0 libraries. More details regarding the implemented algorithm can be found in the aforementioned repository.

The phasogrammetric system was implemented [49] on the basis of two Baumer (Frauenfeld, Switzerland) VCXG-32M video cameras, two VS Technology (Tokyo, Japan) SV-0814H lenses, and a Benq (Taipei, China) W700 projector. The cameras are based on a 1/1.8″ Sony (Tokyo, Japan) IMX265 CMOS sensor with a maximum resolution of 2048 × 1536 pixels, pixel size of 3.45 µm × 3.45 µm, 12-bit resolution of the resulting images, and a Gigabit Ethernet interface connection. The lenses had a focal length of 8 mm and relative aperture of 1:1.4. The projector is based on DLP technology with a resolution of 1280 × 720 pixels, contrast ratio of 10,000:1, and light flux of 2200 lm.

### 2.3. Technique for Estimating the Error of Close-Range Photogrammetric Systems and Algorithms

**Figure 2:**
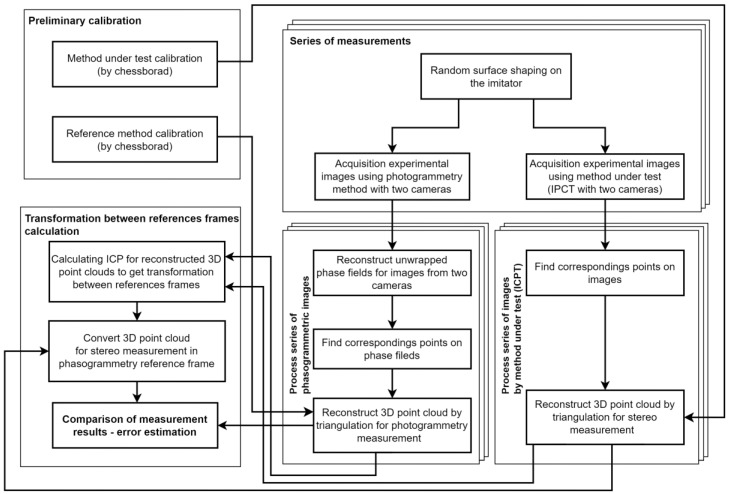
Scheme of the technique of close-range photogrammetric systems error estimation.

The proposed technique (Figure 2) for estimating the error of close-range photogrammetric systems is based on physical modelling using the created system. In order to obtain statistically significant results, it is proposed to measure different (random) surfaces created with the help of a deformable surface imitator. Each surface is measured using a reference method—a phasogrammetric system and a test photogrammetric system. The results of the measurements from the test system are then translated into the coordinate system of the reference system. The results converted into one coordinate system are compared with each other and the difference between them serves as an estimate of the error of the system under test. In this work, all measurements were made in the laser sensor coordinate system. In this system the XOY plane is parallel to the ground plane. The difference between the measurements is the absolute difference between the measured surface height (Z coordinate) of the reference method and the method under test.

(8) $$\left|\Delta Z\left(x,y\right)\right|=\left|Z_{ref}\left(x,y\right)-Z_{test}\left(x,y\right)\right|,$$

where *x*, *y*—surface point coordinates; *Z_ref_*(*x*, *y*)—height measured by reference method; and *Z_test_*(*x*, *y*)—height measured by the test method.

One of the main problems when measuring a three-dimensional point cloud of an object using different devices or methods is how to combine the measurement results. There are two ways to solve this problem: instrumental measurements, which will allow us to measure the parameters of transition from one reference frame to another, and combining the already obtained measurement results using numerical methods. The second method is used in our work. An algorithm to minimize the difference between two clouds of points—Iterative Closest Point (ICP) [50,51]—is used to calculate the parameters of the transition from the coordinate system of the test method to the coordinate system of the reference method. The final parameters are defined as the average value of the parameters determined for the whole series of measurements without taking into account the outliers. To calculate the ICP we used the implementation of the simpleICP library [52,53] in the Python language.

After ICP calculation, the three-dimensional point cloud from the tested method is transformed by the following equation:

(9) $$\left(\begin{array}{c} \begin{array}{c} X{`}_{ref}\\ Y{`}_{ref} \end{array}\\ Z{`}_{ref} \end{array}\right)=[R_{ICP}|t_{ICP}]\left(\begin{array}{c} \begin{array}{c} X_{test}\\ Y_{test} \end{array}\\ Z_{test} \end{array}\right),$$

where *X*`_ref_, *Y*`_ref_, and *Z*`_ref_—coordinates of the surface points obtained by the tested method and transformed by the calculated transformation using the IPC algorithm; **R***_ICP_* and **t***_ICP_—*rotation matrix and translation vector calculated using the ICP algorithm; and *X_text_*, *Y_text_*, and *Z_test_*—coordinates of surface points in the point cloud of the tested method. Since the number of points and their coordinates do not match in the measurements of the reference and tested methods, interpolation for the 3D coordinates of the point cloud with higher density is used to calculate *Z_ref_*(*x*, *y*) and *Z_test_*(*x*, *y*). The final estimation of measurement error is performed using Formula (8).

## 3. Results

### 3.1. Calibration and Verification of the Developed System

**Figure 3:**
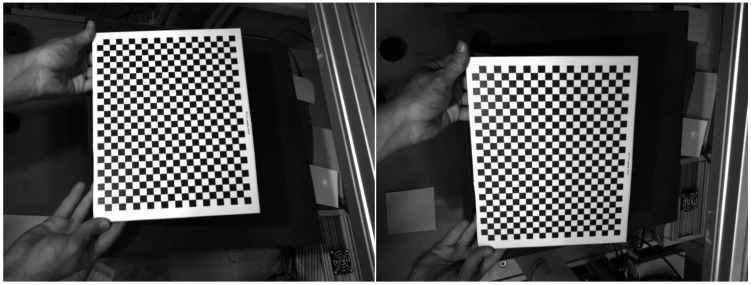
Example of a stereo image pair of a chessboard target used to calibrate the phasogrammetric system.

A classical chessboard target is used to calibrate the phasogrammetric system. The target was fabricated by applying a film with a pattern on a glass substrate from a holographic photographic plate to obtain more accurate calibration results. The target contained 22 vertical and 27 horizontal squares of 10 mm × 10 mm and 21 × 26 points for calibration. Calibration was based on 50 stereo pairs of images of the calibration target at different positions. The search for points in the images and the calculation of the camera and stereo parameters were performed using the OpenCV library for the pinhole model. An example of a stereo image pair of a chessboard target used to calibrate the phasogrammetric system is shown in Figure 3.

During the test phasogrammetric measurements after the initial calibration, it was found that the calculated parameters of the stereo system did not correspond to the real parameters. To verify this, the coordinates *c_x_* and *c_y_* of the intersection of the optical axis of the lens with the CMOS sensor were measured using an AKT-15 autocollimator. The refined values of the centers were fixed by repeated calculations of the stereo system parameters. The parameters CALIB_USE_INTRINSIC_GUESS and CALIB_FIX_PRINCIPAL_POINT were used for the calibration. The resulting camera parameters are listed in Table 1. The final reprojection error of the stereo camera system is 0.220 pixels.

**Figure 4:**
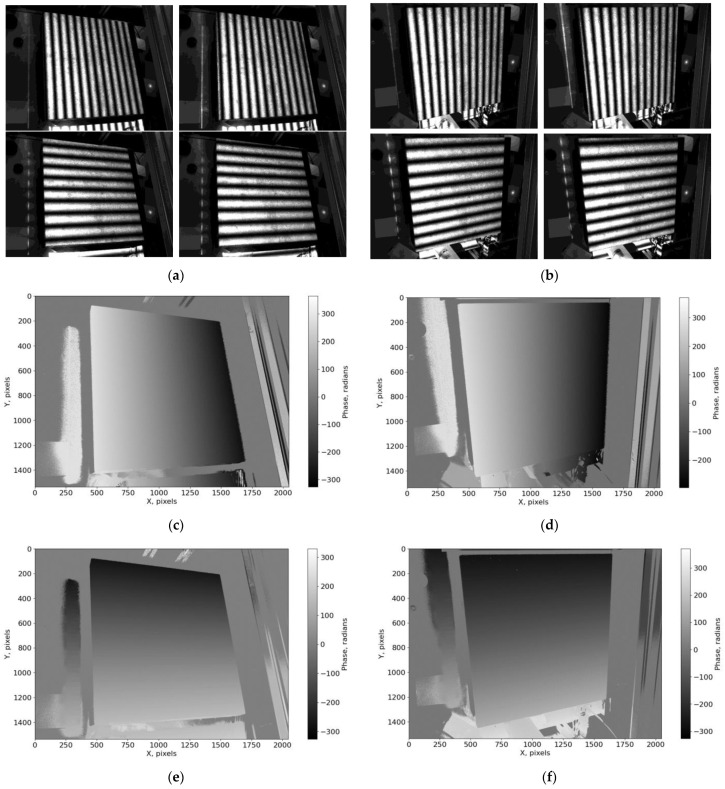
Examples of experimental images and phase fields calculated from them: (**a**) example images with vertical and horizontal fringes for the first camera; (**b**) example images with vertical and horizontal fringes for the second camera; (**c**) horizontal phase field for the first camera; (**d**) horizontal phase field for the second camera; (**e**) vertical phase field for the first camera; (**f**) vertical phase field for the second camera.

To verify the operation of the phasogrammetric system, a series of measurements were performed on the surface of a granite test slab with a non-planarity of no more than 3 µm. The measurements were made using a laser distance sensor and phasogrammetric system. The sensor measured 144 points on a surface of 400 × 400 mm^2^. The measurement time for all the points on the surface was approximately 20 min. The processing of the phasogrammetric images (example images are shown in Figure 4a,b) was performed according to the method described in Section 2.2. Phase fields with discontinuities were calculated for different numbers of fringes, and those without discontinuities were reconstructed using a hierarchical approach (Figure 4c–e). Corresponding points with the same vertical and horizontal phase values were then searched for at a sub-pixel resolution. The final result of the phasogrammetric system measurement is a cloud of 3D points obtained after triangulation.

The measurement error was estimated using the following technique. On the received cloud of 3D points, the coefficients of the plane corresponding to the plane of the granite slab surface were calculated using the least-squares method. The deviation in the measured points from this plane is then calculated. The calculation of the mean square deviation allows the estimation of the measurement error. This technique was applied to the results of both the laser sensor and phasogrammetric system measurements.

**Figure 5:**
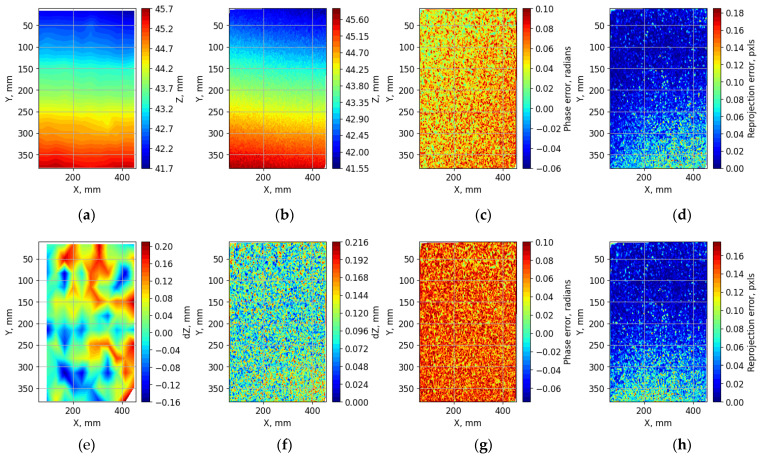
Example of the result of a single measurement of the surface of the granite test slab: (**a**) laser sensor measurement result; (**b**) phasogrammetric system measurement result; (**c**) difference in vertical phase field between corresponding points; (**d**) reprojection error for the phase field from the first camera; (**e**) deviation of the laser sensor measurements from the fitting plane; (**f**) Deviation of the phasogrammetric system measurements from the fitting plane; (**g**) difference in horizontal phase field between corresponding points; (**h**) Reprojection error for the phase field from the second camera.

Figure 5 shows an example of the measurements on a granite slab. The results of the surface measurements are presented in the coordinate system of the laser sensor. The XOY plane was approximately parallel to the surface. The origin is close to the upper-left corner of the surface, and the small value in Y corresponds to the furthest point from the stereo and phasogrammetric cameras. The Z axis is down. Therefore, a small value of Z corresponds to the highest points on the surface relative to the table surface. The measurement of a surface for the phasogrammetric system contains approximately 27,000 points. In the example shown in Figure 5, the RMS errors for the sensor was 39.2 µm and was 46.6 µm for the phasogrammetric system. The RMS value of the re-projection error for the latter was 0.0328 pixels.

**Figure 6:**
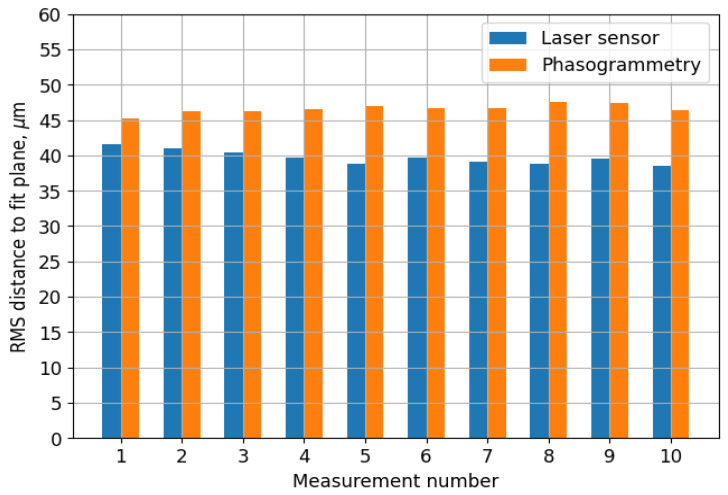
Results of a series of measurements of the surface of the granite test slab; comparison of the distance standard deviation from the fitting plane for the laser sensor and the phasogrammetric system.

The results of the entire series of measurements on the granite slab are shown in Figure 6. It is clear from the results that the measurement error of the laser sensor is smaller than that of the phasogrammetric system. However, the absolute difference is only 5–9 µm. Simultaneously, the phasogrammetric measurement was 20–40 times faster and did not cause any additional vibrations. Figure 6 also shows that the measurement system has a high stability, and the measurement error does not depend on the time for at least 3 h.

### 3.2. Testing of the Stereo Photogrammetric System

In this study, the Image Pattern Correlation Technique (IPCT) [17] was chosen as the testing method. It is a variant of the Digital Image Correlation (DIC) method [54] that uses algorithms from the Particle Image Velocimetry (PIV) method [55] for processing. In the IPCT, a special background pattern consisting of randomly placed black dots on a white background was applied to the surface prior to measurement. Stereo images of the surface were captured using two cameras to determine the shape of the surface. Processing involves dividing the stereo images into interrogation windows and calculating the correlation function between the corresponding windows. The maximum of the correlation function makes it possible to determine the displacement of the interrogation windows relative to each other and, on this basis, to determine the corresponding points for which a triangulation is calculated to estimate the three-dimensional coordinates of the surface. In addition, ArUco fiducial markers [56], which are also applied to the measured surface, are used for initial image matching [57].

**Figure 7:**
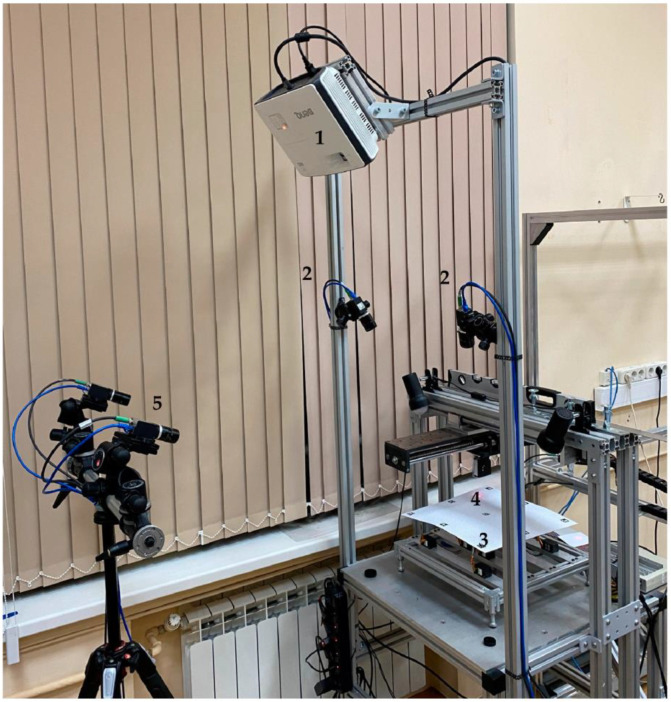
Error estimation system for close-range photogrammetric systems with tested stereo photogrammetric system (1—projector; 2—photogrammetric system cameras; 3—imitator of the deformable surface; 4—optical distance sensor; 5—tested stereo photogrammetric system).

The tested stereo photogrammetric system was realized based on two Baumer VCXG-32M cameras (whose technical parameters are given in Section 2.2) and VST SV-1614H lenses with a focal length of 16 mm and a relative aperture of 1:1.4. Figure 7 shows a general view of the measurement error estimation system with the tested photogrammetric system. As the IPCT was originally designed for flight testing, the position of the stereo system relative to the measured surface was chosen to be similar to experimental studies where the angle between the optical axis and the measured plane is quite small. In the experiment, the angle was approximately 25°. Prior to the tests, the camera stereo system was calibrated using the same technique (Section 3.1) as the phasogrammetric system. The parameters obtained for the stereo system are presented in Table 2.

**Figure 8:**
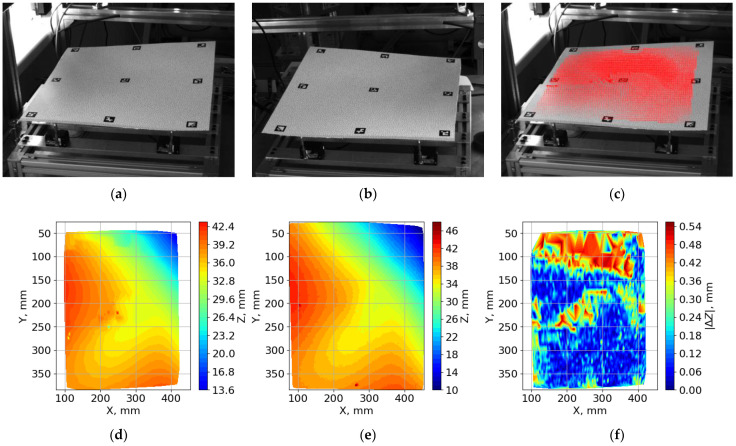
Example of the result of processing of one random surface generated on the imitator: (**a**) image from the first camera of the stereo system; (**b**) image from the second camera of the stereo system; (**c**) vector field obtained by cross-correlation processing of a stereo pair of images; (**d**) result of surface reconstruction by the stereo photogrammetric system; (**e**) result of surface reconstruction by the phasogrammetric system; (**f**) absolute difference between the results of measurements along the *z*-axis.

In the experiment, 100 random surface shapes were measured on the imitator using the phasogrammetric and stereo photogrammetric methods (IPCT). Error estimation was performed according to the method described in Section 2.3. Figure 8a,b shows an example of a stereo image pair obtained during the experiment. The images were processed using a single-pass calculation of the cross-correlation function for interrogation windows of 128 × 128 pixels, with a step of 16 pixels vertically and horizontally. The total number of 3D points calculated for IPCT measurement was approximately 3000. Gaussian function interpolation was used to determine the maximum correlation with sub-pixel accuracy. This approach is widely used in image processing for the PIV method, the algorithms of which are used in the IPCT method. A brief explanation is that a well-focused point in an image is represented by an Airy circle, which in turn, is well-approximated by a Gaussian function [55]. Filtering of invalid vectors was not specifically used to obtain more pronounced errors in surface reconstruction and to obtain results characteristic of the IPCT image processing. An example of the calculated vector field is shown in Figure 8c.

A comparison of the surface shape measurement results from the stereo photogrammetric and phasogrammetric systems is shown in Figure 8d–f. The reconstructed surface area for the IPCT was smaller than that for the phasogrammetric system. This is explained by the fact that the coordinates of the region of interest for the vector field calculation are chosen by the centers of the ArUco markers located at the edges of the surface. The markers were located with a small offset from the surface boundary. Figure 8f shows that the main errors in surface matching occur in the center and upper parts of the surface (the edge of the surface far from the cameras).

**Figure 9:**
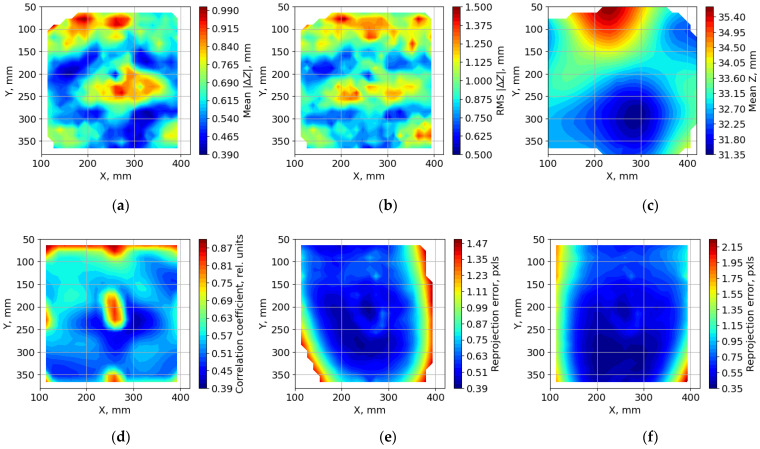
Results of processing a series of measurements of random surfaces formed on the imitator: (**a**) mean value of the absolute difference between stereo photogrammetric and phasogrammetric measurements; (**b**) standard deviation of the absolute difference between stereo photogrammetric and phasogrammetric measurements; (**c**) mean value of the surface height; (**d**) mean value of the maximum of the correlation function in the interrogation windows for IPCT; (**e**) mean value of the reprojection error for the first camera of the stereo photogrammetric system; (**f**) mean value of the reprojection error for the second camera of the stereo photogrammetric system.

Figure 9 summarizes the results of processing a series of measurements for 100 random surfaces generated on the imitator. For reference, Figure 9c shows the average surface height for all 100 measurements. It can be seen that it varies over the entire surface within a range of only 4 mm, with measured surface heights ranging from 10 to 60 mm for all measurements. Figure 9a,b shows that the measurement difference increases at the center and top of the surface. The standard deviation in the measurement difference also increased in these areas as well as in the corners of the surface. The increase in error at the top edge can be explained by the decrease in spatial resolution in this region of the image because this edge is at a greater distance from the cameras. A theoretical estimation of the spatial resolution (Equation (9)) for a measured surface at a small angle to the optical axis of the video camera is given in [58]. Calculations using the formula show that the spatial resolution of the image at the near edge of the surface will be approximately 1.62 pxl/mm and would be 0.931 pxl/mm at the far edge. The reduction in this value by almost a factor of two can explain the increase in the measurement error of the far edge of the surface.

To explain the error in the center, we considered the intermediate processing results for the IPCT. Figure 9d shows the distribution of the mean values of the maximum correlation function for the interrogation windows obtained during the processing of the experimental images. The value of the maximum correlation for the IPCT serves as an estimate of the reliability of the displacement value. The higher the value, the better the fit of the displaced interrogation windows. Therefore, all other things being equal, a high value for the maximum correlation function is expected to result in more accurate measurements. High correlation values were observed at the center, corners, and edges of the surface far from the cameras. That is, in places where the difference in surface measurements is maximum.

This inconsistency could be explained by the presence of ArUco markers on the surface. They represent rather large areas of black image intensity compared with the individual points of the background pattern. This is why there was an almost completely black square in the interrogation window. This results in a high value of the correlation function, even if the displacement is incorrectly determined. The upper edge of the plane had a high function value for the same reason. The only difference is that the interrogation window includes a monotonous part of the image intensity that lies outside the boundaries of the imitation surface.

During processing, the preliminary displacement of the stereo pairs was performed using four markers located at the corners of the surface. Therefore, the displacements between the combined stereo pairs are minimal at these locations. The further away from the corners, the greater the differences between the combined images, which causes the average value of the maximum correlation function to decrease from the corners to the center.

Figure 9e,f shows the distribution of the mean values of the reprojection error for the cameras of the stereo photogrammetric system. This value is one of the main ways of assessing the accuracy of measurements in methods that use triangulation to obtain three-dimensional coordinates from a set of two-dimensional coordinates. At the edges of the surface, the reprojection error increases sharply, which can be explained by the edge effects when the interrogation windows fall on the edges of the surface. In the central region, the reprojection error is in the range of up to 1 pixel. In general, the distribution was similar to the distribution of the difference between the measurement results, but not completely. The distribution of the reprojection error in the central region does not fully correspond to the increasing difference between the measurements.

## 4. Discussion

This study proposes an approach to estimate the error of photogrammetric systems based on physical modelling. For this purpose, a system based on a deformable surface imitator is developed, which allows the creation of surfaces of different shapes and measurement by photogrammetry and other reference methods with higher accuracy. Phasogrammetry, which is a mixture of stereo photogrammetry and phase-shift profilometry, was chosen as the reference method.

The phasogrammetric system was based on two machine-vision video cameras and a computer projector. Python software [48] was used to generate projected patterns, display them on the projector, capture surface images with the video cameras, and process them. Quantitative tests of the phasogrammetric system on a granite test slab showed that the system had an error of approximately 50 µm for a measured surface size of 400 × 400 mm^2^.

To verify the performance of the developed system, a series of random surface shapes measured by the phasogrammetric system and stereo camera system using IPCT were compared. The experiment demonstrated the possibility of using the proposed measurement-error estimation technique. The aggregated results of the series of measurements helped to identify an increase in the measurement error at the center of the surface caused by the presence of the ArUco marker. This feature was not visible in the individual measurements. The results obtained were compared with values often used to indirectly evaluate the results of 3D surface measurements: the reprojection error and the maximum correlation function for the IPCT method. The experimental results showed that these values do not always provide a reliable estimate of measurement accuracy. Therefore, physical modelling using the proposed technique may reveal dependencies that cannot be determined using other techniques.

The main disadvantages of the proposed system and the proposed methodology are the direct dependence of the results on the error of the reference method, the necessity of combining the measurements of the reference and tested methods in one coordinate system, the complexity of adjusting the measuring system for different measurement scales, and the requirement of the physical presence of an experimental system. The measurement accuracy achieved in the phasogrammetric system is similar to that of other analogous works [43,44,59]. In future work, the accuracy and other parameters of the system can be improved in various ways, such as the modernization of the hardware component of the system, the use of the 12-bit mode in the video camera acquisition, the improvement of the algorithm when searching for points with the same phase, the implementation of the auto-calibration algorithm [44] to refine the calibration parameters of the video cameras of the phasogrammetric system, the consideration of the reflectivity of the measured surfaces [60], and the use of modern modifications of the ICP algorithm [61], including the search for a global minimum.

Thus, the first two disadvantages can be partially compensated by the above mentioned approaches. However, even at the current level, the estimation can be applied to another class of methods and devices that do not require the high accuracy of the reference method. Examples of such applications are the error estimation of RGB-D cameras and stereo cameras for robotics based on disparity calculations.

The created system can be adjusted to the measurement scale by changing the field of view of the cameras of the phasogrammetric system, and by changing the position and amplitude of the servo movement. However, the maximum change in the scale can be estimated up to 3–5 times. A larger change may require significant changes in the hardware components of the system.

The last disadvantage is difficult to eliminate because the system is not mobile. However, the main purpose of this system is to perform fundamental research to evaluate the influence of various factors on the measurement error of photogrammetric methods or algorithms, and to evaluate the error of various off-the-shelf devices, such as RGB-D cameras.

The main advantage of the created system is the possibility of considering the majority of parameters influencing the measurement error and the possibility of estimating the influence of different factors on the measurement error separately based on physical simulation. For example, the possibility of estimating the influence of measured surface illumination or surface profile height on the measurement results of a particular photogrammetric method or device.

Let us consider the proposed method and the system created in the context of existing error estimation methods for close-range photogrammetry. If we compare them with theoretical estimation methods (e.g., refs. [27,29]), the latter can be applied to ready-made test setups to estimate instrumental error. Their main idea was to calculate the measurement uncertainty through stereo vision calibration and measurement algorithms. In this case, it is impossible to consider the many factors that occur in a real experiment. If we look at studies on error estimation based on the processing of synthesized images, for example [28], this approach also has its limitations. Even photorealistic images may not correspond to the physical dimensions of objects specified in the simulation.

Experimental methods use test objects of known dimensions, typically ceramic spheres (e.g., ref. [44]) or flat plates (e.g., ref. [45]), which are moved into the field of view using linear translators. The dimensions of the former are usually much smaller than the field of view of the photogrammetric systems under test, and the flat plate surface is too simple to evaluate the error fully. Neither type of object allows the error of the tested systems to be fully determined by considering possible nonlinearity and in the entire measurement volume. Another possibility is a direct comparison of the measurement results of the method under test with those of another method, e.g., ref. [33]. However, usually, only one or several measured objects are used, and it is impossible to estimate the full error by comparing them. The proposed method is an attempt to combine two approaches of experimental methods: the use of a method with known and relatively high measurement accuracy and an imitator of a deformable surface, which allows a large number of objects to be obtained for the comparison of measurement results.

Further work will be aimed at improving the accuracy of the 3D point cloud matching obtained by the reference and tested systems and implementing an autocalibration algorithm for the phasogrammetric system. The practical application of the developed system is aimed at conducting experimental studies to estimate the influence of changing the effective calibration parameters of the camera stereo system on the results of photogrammetric measurements.

## Figures and Tables

**Table 1:** Stereo calibration parameters for the phasogrammetric system cameras.

Parameter	Camera 1	Camera 2
Focus length (*f_x_*, and *f_y_*)	2325.149	2328.429
	2333.059	2336.481
Principal point, (*c_x_* and *c_y_*)	979	1047
	789	814
Distortion coefficients	−0.0151538	0.0260202
	−0.0458932	0.0533106
	0.0003807	0.0002164
	0.0001624	0.0007239
	0.3395086	0.0152157
Reprojection error, pxl	0.198	0.175
Rotation matrix between cameras (**R**)	$\left[\begin{array}{ccc} 0.99580 & 0.04637 & 0.07900\\ -0.08268 & 0.82622 & 0.55724\\ -0.03943 & -0.56143 & 0.82658 \end{array}\right]$	
Translation vector between cameras (**t**), mm	$\left[\begin{array}{ccc} 15.887 & 403.947 & 124.193 \end{array}\right]$	
Stereo reprojection error, pxl	0.220	

**Table 2:** Stereo calibration parameters for the photogrammetric system cameras.

Parameter	Camera 1	Camera 2
Focus length (*f_x_*, and *f_y_*)	4707.373	4734.902
	4692.853	4718.614
Principal point, (*c_x_* and *c_y_*)	1024	1024
	768	768
Distortion coefficients	−0.13454450	−0.12423092
	0.17658227	−0.30509181
	−0.00137111	−0.00108892
	−0.00023123	−0.00123370
	6.38129081	11.63218979
Reprojection error, pxl	0.177	0.186
Rotation matrix between cameras (**R**)	$\left[\begin{array}{ccc} 0.92927 & -0.06228 & 0.36411\\ 0.06338 & 0.99795 & 0.00893\\ -0.36392 & 0.01478 & 0.93121 \end{array}\right]$	
Translation vector between cameras (**t**), mm	$\left[\begin{array}{ccc} 406.209 & 4.520 & 55.758 \end{array}\right]$	
Stereo reprojection error, pxl	0.206	

## Data Availability

The data presented in this study are available on request from the corresponding author.
